# Association between prediabetes and the incidence of gastric cancer: A meta-analysis

**DOI:** 10.1097/MD.0000000000039411

**Published:** 2024-08-23

**Authors:** Shenggang Wang, Jiamin Zhao, Chong Liu

**Affiliations:** a Department of Gastrointestinal Surgery, Weifang People’s Hospital, Weifang, China; b Department of Urology Surgery, Weifang People’s Hospital, Weifang, China.

**Keywords:** dysglycemia, gastric cancer, meta-analysis, prediabetes, risk

## Abstract

**Background:**

Prediabetes has been found to be associated with an elevated overall risk of cancer, which may be site-specific. we performed a protocol for systematic review and meta-analysis to investigate the correlation between prediabetes and the incidence of gastric cancer (GC).

**Methods:**

A thorough review of the literature was conducted in the PubMed, Embase, and Web of Science databases to identify pertinent observational studies with longitudinal follow-up. The random-effects model was employed to consolidate the data, taking into account the potential impact of heterogeneity.

**Results:**

A total of 13 datasets from 8 prospective cohort studies were included. The prevalence of prediabetes was 9.6%. During the mean follow-up duration of 7.1 to 12.2 years, 33,135 patients were diagnosed with GC. According to the results of the pooled analysis, prediabetes was associated with a mildly higher incidence of GC over time (risk ratio: 1.07, 95% confidence interval: 1.01–1.13, *P* = .03; *I*^2^ = 44%). Subsequent subgroup analyses indicated that the relationship between prediabetes and the heightened risk of GC may not be substantially influenced by factors such as the country in which the study was conducted, the average age of participants, their gender, the definition of prediabetes used, the prevalence of prediabetes at the beginning of the study, the incidence of GC within the studied population, or the adjustment made for body mass index (*P* for subgroup difference all >.05).

**Conclusion:**

The presence of prediabetes may increase the risk of GC by a mild amount when compared with people with normoglycemia in community-derived adult populations.

## 1. Introduction

Gastric cancer (GC) is a common malignancy of digestive system.^[1,2]^ According to the epidemiological data, more than 1 million new cases of GC were diagnosed in 2020, and 768,793 people died of GC globally in this year.^[3,4]^ Due to the aging of the global population and growth of high-risk groups, number of patients with GC worldwide is expected to continue growing in the future.^[5]^ Although comprehensive treatments such as surgical resection, chemoradiotherapy, and immunotherapy etc have been well used for patients with GC, the prognosis of these patients, particularly for those with advanced stages, remains poor.^[3,6]^ Accordingly, it is important to determine the reversible risk factor for GC, which may be of significance to prevent the disease.

Diabetes is a prevalent metabolic disorder, which has been related to various cardiovascular complications.^[7]^ The incidence of diabetes is expected to continuously increase, even in special population such as peritoneal dialysis patients.^[8]^ Diabetes has been related to multiple cardiovascular and renal complications, which may be mediated by hyperglycemia, insulin resistance, and perhaps genetic factors.^[9]^ In addition, accumulating evidence has also linked hyperglycemia to cancers.^[10]^ A recent meta-analysis in 2022 involving 40 cohort studies suggested that both type 1 and type 2 diabetes confer a higher risk of GC,^[11]^ suggesting that hyperglycemia may be involved in the pathogenesis of GC. Moreover, it has been proposed that prediabetes, which serves as a transitional phase between normoglycemia and diabetes, may exhibit a heightened susceptibility to cancer on a broader scale.^[12]^ Prediabetes can be clinically characterized by the presence of impaired fasting glucose (IFG), impaired glucose tolerance (IGT), and mildly elevated glycated hemoglobin (HbA1c).^[13,14]^ While a previous meta-analysis has demonstrated an overall increased cancer risk in individuals with prediabetes, the association may vary depending on the particular cancer site.^[15]^ Nevertheless, previous studies examining prediabetes and incidence of GC have reached inconsistent results.^[16–23]^ Therefore, the aim of the systematic review and meta-analysis was to examine in detail the association between prediabetes and GC risk in community-derived populations.

## 2. Materials and methods

The study conformed with the guidelines of the Preferred Reporting Items for Systematic Reviews and Meta-Analyses statement^[24,25]^ and the Cochrane Handbook of Systematic Reviews of Interventions.^[25]^

### 2.1. Inclusion and exclusion criteria of studies

The inclusion criteria were formulated based on the Population, Intervention, Comparison, Outcomes, and Study (PICOS) design guidelines, as well as aligned with the objective of the meta-analysis.

P (participants): Community derived adult population without confirmed diagnosis of GC at baseline. We did not restrict the geographical locations in the inclusion criteria or search terms. Similarly, no restriction has been applied regarding the population in the search strategy. Only community derived population was considered because this population is of the representative significance for the topic of our meta-analysis for the evaluation of GC risk factors.

I (exposure): Participants with prediabetes, which was defined in accordance with the criteria used among the include studies involving the current definition for IFG, IGT, and mildly elevated HbA1c.^[13,14]^

C (control): Participants with normoglycemia.

O (outcomes): Compared the incidence of GC between participants with prediabetes and those with normoglycemia, which was presented as risk ratio (RR) with 95% confidence interval (CI), or these data could be estimated. The methods for the confirmation of patients who developed GC during follow-up were also consistent with the methods used in the original studies.

S (study design): Observational studies with longitudinal follow-up, which included cohort studies, post hoc analyses of clinical trials, and nested case–control studies.

Other study types such as reviews, editorials, letters, and meta-analyses were excluded. Furthermore, studies were excluded if they enrolled patients with GC at baseline, did not evaluate prediabetes as exposure, did not report the incidence of GC during follow-up, or did not provide adequate data to generate RR and corresponding 95% CI. In cases where there was overlap in the populations being studied, the meta-analysis incorporated the study with the largest sample size.

### 2.2. Search of databases

A comprehensive search was conducted in electronic databases including PubMed, Embase, and Web of Science, from their inception until September 27, 2023, to identify studies published within this timeframe. The search strategy involved combining the terms of (1) “prediabetes” OR “prediabetic” OR “pre-diabetes” OR “pre-diabetic” OR “borderline diabetes” OR “prediabetic state” OR “impaired fasting glucose” OR “IFG” OR “impaired glucose tolerance” OR “IGT” OR “HbA1c”OR “fasting glucose”; (2) “gastric” OR “stomach”; and (3) “neoplasms” OR “cancer” OR “tumor” OR “carcinoma” OR “adenoma” OR “malignancy.” The detailed search strategy was provided in Supplemental Material S1, Supplemental Digital Content, http://links.lww.com/MD/N416. The analysis included only full-length articles published in peer-reviewed journals in English language. Additionally, relevant review and original articles were manually screened for potential studies of interest.

### 2.3. Data extraction and quality evaluation

Literature searches, data collection, and study quality assessments were independently carried out by 2 authors. In instances of disparities, a third author was enlisted to facilitate discourse and attain unanimity. Concerning the studies incorporated in the analysis, we meticulously gathered comprehensive data encompassing study characteristics, general status of the encompassed population, participant quantities, average age, gender distribution, diagnostic criteria for prediabetes, durations of follow-up, and methodologies employed to confirm instances of GC. Additionally, we recorded the variables that were adjusted for during the analysis of the association prediabetes and the incidence of GC. To evaluate the quality of each study, we used the Newcastle–Ottawa Scale, which assesses based on criteria such as participant selection, group comparability, and the validity of the outcomes. This scale awards up to 9 stars, with a higher score indicating a more rigorous study. The participant selection was considered to be with adequate quality if it is consecutively or randomly enrolled. The exposure (prediabetes) is considered to be adequately defined and evaluated if it was consistent with the current definition for IFG, IGT, and mildly elevated HbA1c.^[13,14]^ The assessment of GC outcome was considered to be adequately defined if it is diagnosed with clinical evaluation incorporating pathological examinations. The follow-up duration was considered to be enough long if the mean follow-up duration is at least for 5 years.

### 2.4. Statistics

The current study utilized RRs and their corresponding 95% CI to summarize the association prediabetes and the risk of GC during follow-up. Whenever feasible, the RR and CI obtained from the regression model with the most comprehensive adjustment were extracted. In order to achieve stability and normalize variance, a logarithmic transformation was applied to the RR and its corresponding standard error in each study.^[25]^ To evaluate heterogeneity between studies, the Cochrane Q test and the I^2^ statistic^[26]^ were employed, with an I^2^ value exceeding 50% indicating substantial heterogeneity. The results were combined using a random-effects model, which has been recommended for accounting for potential heterogeneity between studies.^[25]^ Sensitivity analysis by excluding one dataset at a time was performed to evaluate the robustness of the finding.^[25]^ The influences of study characteristics in continuous variables, such as mean age, proportion of men, and follow-up duration were evaluated in univariate meta-regression analysis.^[25]^ Subgroup analyses were conducted to assess the impact of predefined study characteristics on the outcome, such as study country, design, age, sex, definition of prediabetes, prevalence of prediabetes at baseline, incidence of GC of the studied population, and adjustment of body mass index (BMI). Publication bias was assessed using a funnel plot and Egger regression asymmetry test, which relied on visual symmetry judgments.^[27]^ Statistical analyses were performed using RevMan (Version 5.1; Cochrane Collaboration, Oxford) and Stata software (Version 12.0; Stata Corporation, College Station, TX).

## 3. Results

### 3.1. Literature search and study retrieval

**Figure 1.**
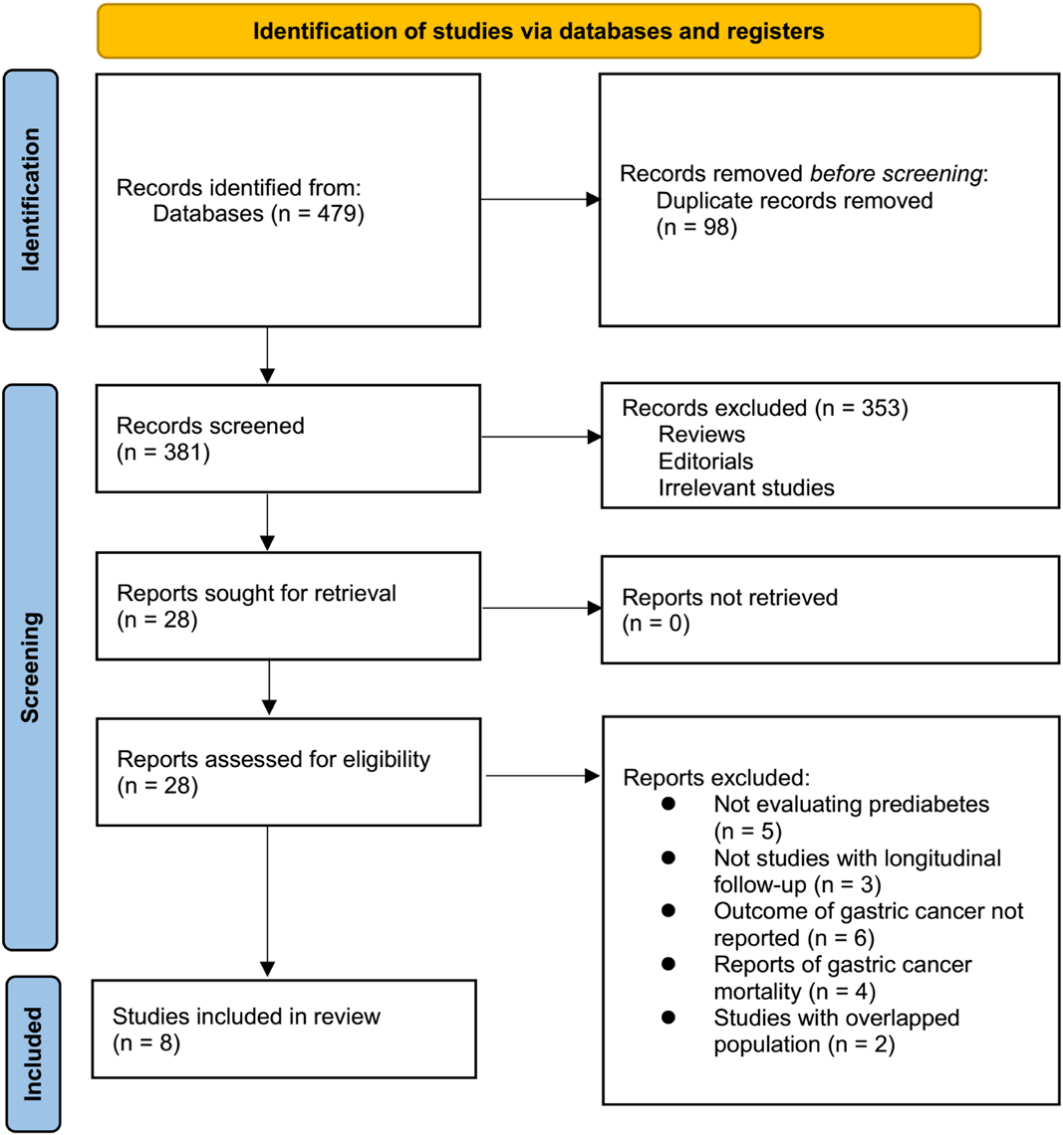
Flowchart of database search and study inclusion.

Figure 1 depicts the sequential steps involved in the literature search and study retrieval process. Initially, a total of 479 records were obtained through the database search. Subsequently, 98 duplicate entries were identified and eliminated. Upon scrutinizing the titles and abstracts, 353 studies were excluded due to their lack of alignment with the meta-analysis objectives. Further examination of the full texts of the remaining 28 studies resulted in the exclusion of an additional 20 studies, with the specific rationales for exclusion outlined in Figure 1. Ultimately, a total of 8 studies were chosen for inclusion in the final meta-analysis.^[16–23]^

### 3.2. Study characteristics

Overall, 8 cohort studies,^[16–23]^ all of prospective design, were included in the meta-analysis. Five of them reported the association between prediabetes and the incidence by gender, and these datasets were included in the meta-analysis separately.^[16–18,22,23]^ The characteristics of the included studies are summarized in Table 1. These studies were published between 2005 and 2023 and were performed in Korea, Austria, Japan, Sweden, the United Kingdom, and China. All of the studies included community derived population, with a total of 7,372,277 participants included. The mean ages of the subjects varied between 43.0 to 57.8 years. Prediabetes was diagnosed with IFG in 4 studies,^[16,17,22,23]^ with IGT in one study,^[19]^ with IFG and/or IGT in 2 studies,^[18,21]^ and with mildly elevated HbA1c in another.^[20]^ Accordingly, 704,719 (9.6%) subjects had prediabetes at baseline. During the mean follow-up duration of 7.1 to 12.2 years, 33,135 patients were diagnosed as GC. The confirmation of the diagnosis of GC was all via the data of local or national cancer registry databases. In the included studies, variables such as age, sex, smoking, alcohol consumption, and BMI were adjusted to varying degrees. Studies included in the analysis received all 8 stars on Newcastle–Ottawa Scale, which indicated that they were high quality studies (Table 2). All of the included studies were scored as 8 with one point deduced for the domain of assessment of outcome because in all of the included studies, GC was diagnosed with cancer registration data rather than clinical evaluation records with pathological examinations.

**Table 1:** Characteristics of the included studies.

Study	Location	Design	Population	No. of participants	Mean age (years)	Men (%)	Diagnosis of PreD	No. of participants with PreD	Follow-up duration (years)	Number participants with GC during follow-up	Methods of GC validation	Variables adjusted
Jee 2005^[16]^ men	Korea	PC	Community population aged 30 or older	829,770	45.3	100	IFG	58,020	10	1111	National cancer registry	Age, smoking, and alcohol use
Jee 2005^[16]^ women	Korea	PC	Community population aged 30 or older	468,615	49.6	0	IFG	22,578	10	257	National cancer registry	Age, smoking, and alcohol use
Rapp 2006^[17]^ men	Austria	PC	Community adult population	63,585	43	100	IFG	3467	8.4	121	National cancer registry	Age, smoking, occupational group, BMI, and alcohol use
Rapp 2006^[17]^ women	Austria	PC	Community adult population	77,228	43	0	IFG	3320	8.4	98	National cancer registry	Age, smoking, occupational group, BMI, and alcohol use
Inoue 2009^[18]^ men	Japan	PC	Community women aged 40 to 69 years	9548	56.5	100	IFG and/or IGT	2158	10.1	233	National cancer registry	Age, study center, smoking, alcohol drinking, and TC
Inoue 2009^[18]^ women	Japan	PC	Community women aged 40 to 69 years	18,176	55.5	0	IFG and/or IGT	2163	10.1	138	National cancer registry	Age, study center, smoking, alcohol drinking, and TC
Zhang 2019^[19]^	Sweden	PC	Community adult population	111,198	49.8	49.3	IGT	23,900	12.2	219	National cancer registry	Age, sex, calendar year, BMI, smoking, and education level
Peila 2020^[20]^	UK	PC	Community population aged from 40 to 69 years	502,536	56.4	54.4	HbA1c 5.7–6.4%	64,167	7.1	380	National cancer registry	Age, sex, education, race, smoking, alcohol use, BMI, and physical activity
Ke 2021^[21]^	China	PC	Community adult population	9224	57.8	36.8	IFG and/or IGT	1454	7.5	44	Local cancer registration and management system	Age, sex, marriage status, education, BMI, smoking, alcohol consumption, tea consumption, physical activity, family history of cancer, history of hypertension, dyslipidemia, viral hepatitis, chronic atrophic gastritis, use anti-inflammatory agents, and serum CRP
Huang 2023^[22]^ men	Korea	PC	Community population aged 40 to 69 years	37,350	52.9	100	IFG	12,493	9.1	408	National cancer registry	Age, smoking, alcohol consumption, family history of cancer, education, physical activity and energy intake
Huang 2023^[22]^ women	Korea	PC	Community population aged 40 to 69 years	71,047	52.7	0	IFG	13,187	9.1	351	National cancer registry	Age, smoking, alcohol consumption, family history of cancer, education, physical activity and energy intake
Nam 2023^[23]^ men	Korea	PC	Community population aged 40 years or older	2,229,627	56	100	IFG	233,161	9	18,241	National cancer registry	Age, smoking, alcohol intake, BMI, and family history of GC
Nam 2023^[23]^ women	Korea	PC	Community population aged 40 years or older	2,944,373	56	0	IFG	264,651	9	11,534	National cancer registry	Age, smoking, alcohol intake, BMI, and family history of GC

BMI = body mass index; CRP = C-reactive protein; GC = gastric cancer; HbA1c = hemoglobin A1c; IFG = impaired fasting glucose; IGT = impaired glucose tolerance; PC = prospective cohort; PreD = prediabetes; TC = total cholesterol.

**Table 2:** Study quality evaluation via the Newcastle-Ottawa scale.

Study	Representativeness of the exposed cohort	Selection of the non-exposed cohort	Ascertainment of exposure	Outcome not present at baseline	Control for age and sex	Control for other confounding factors	Assessment of outcome	Enough long follow-up duration	Adequacy of follow-up of cohorts	Total
Jee 2005^[16]^	1	1	1	1	1	1	0	1	1	8
Rapp 2006^[17]^	1	1	1	1	1	1	0	1	1	8
Inoue 2009^[18]^	1	1	1	1	1	1	0	1	1	8
Zhang 2019^[19]^	1	1	1	1	1	1	0	1	1	8
Peila 2020^[20]^	1	1	1	1	1	1	0	1	1	8
Ke 2021^[21]^	1	1	1	1	1	1	0	1	1	8
Huang 2023^[22]^	1	1	1	1	1	1	0	1	1	8
Nam 2023^[23]^	1	1	1	1	1	1	0	1	1	8

### 3.3. Meta-analysis results

**Figure 2.**
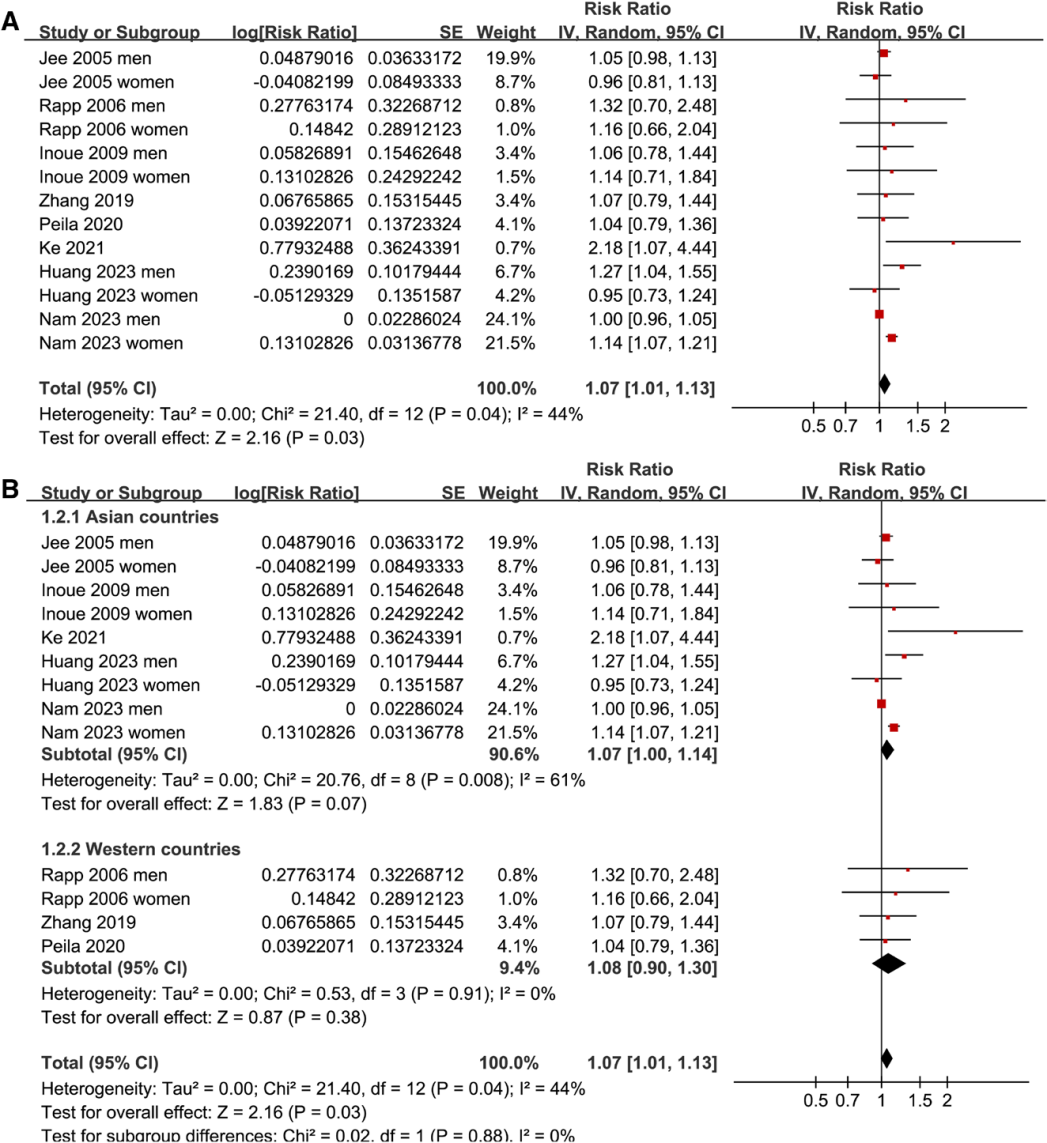
Forest plots for the meta-analysis of the association between prediabetes and the risk of GC; (A) forest plots for the overall meta-analysis; and (B) forest plots for the subgroup analysis according to study country. GC = gastric cancer.

**Figure 3.**
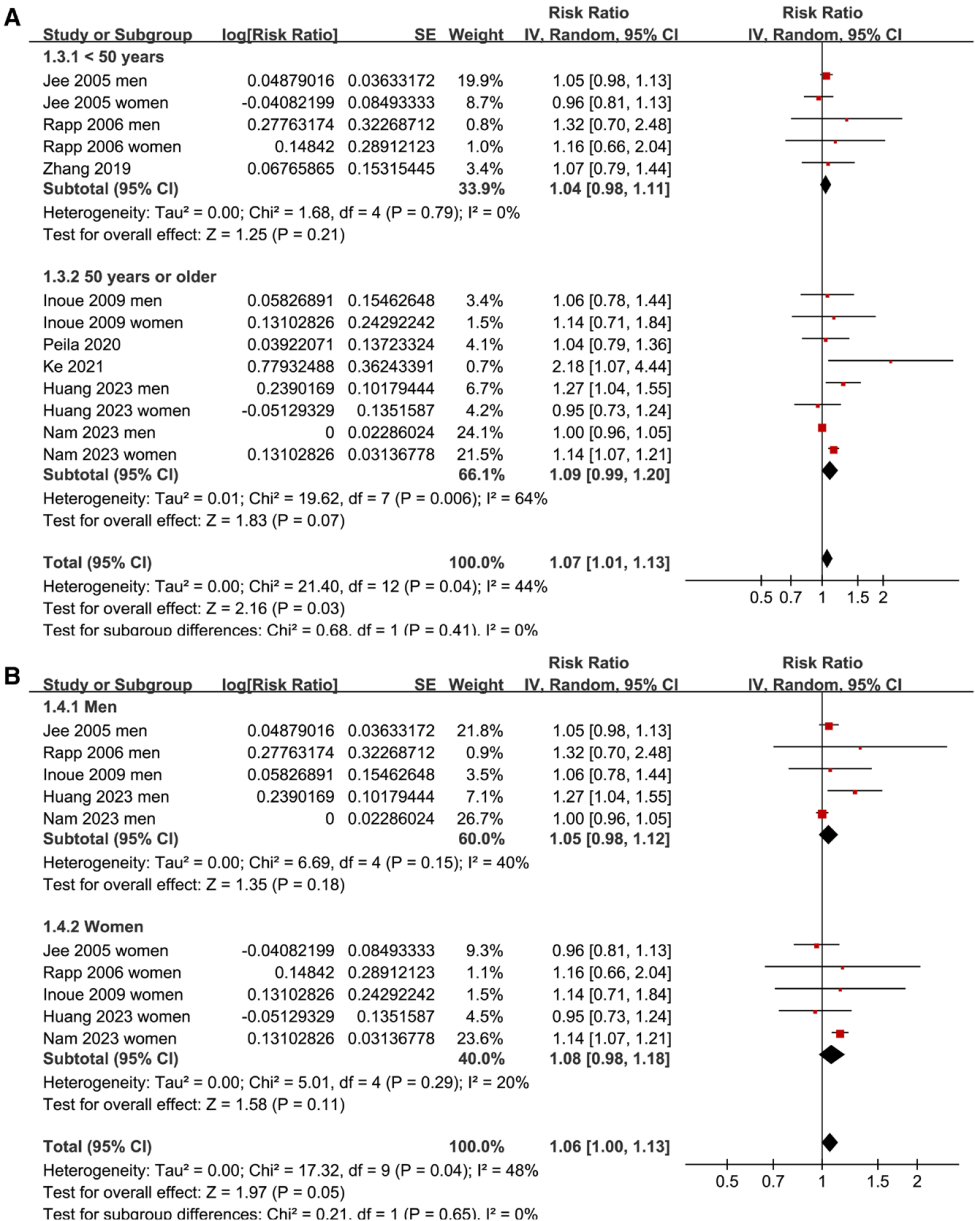
Forest plots for the subgroup analyses of the association between prediabetes and the risk of GC; (A) forest plots for the subgroup analysis according to mean age of the population; and (B) forest plots for the subgroup analysis according to sex of the subjects. GC = gastric cancer.

**Figure 4.**
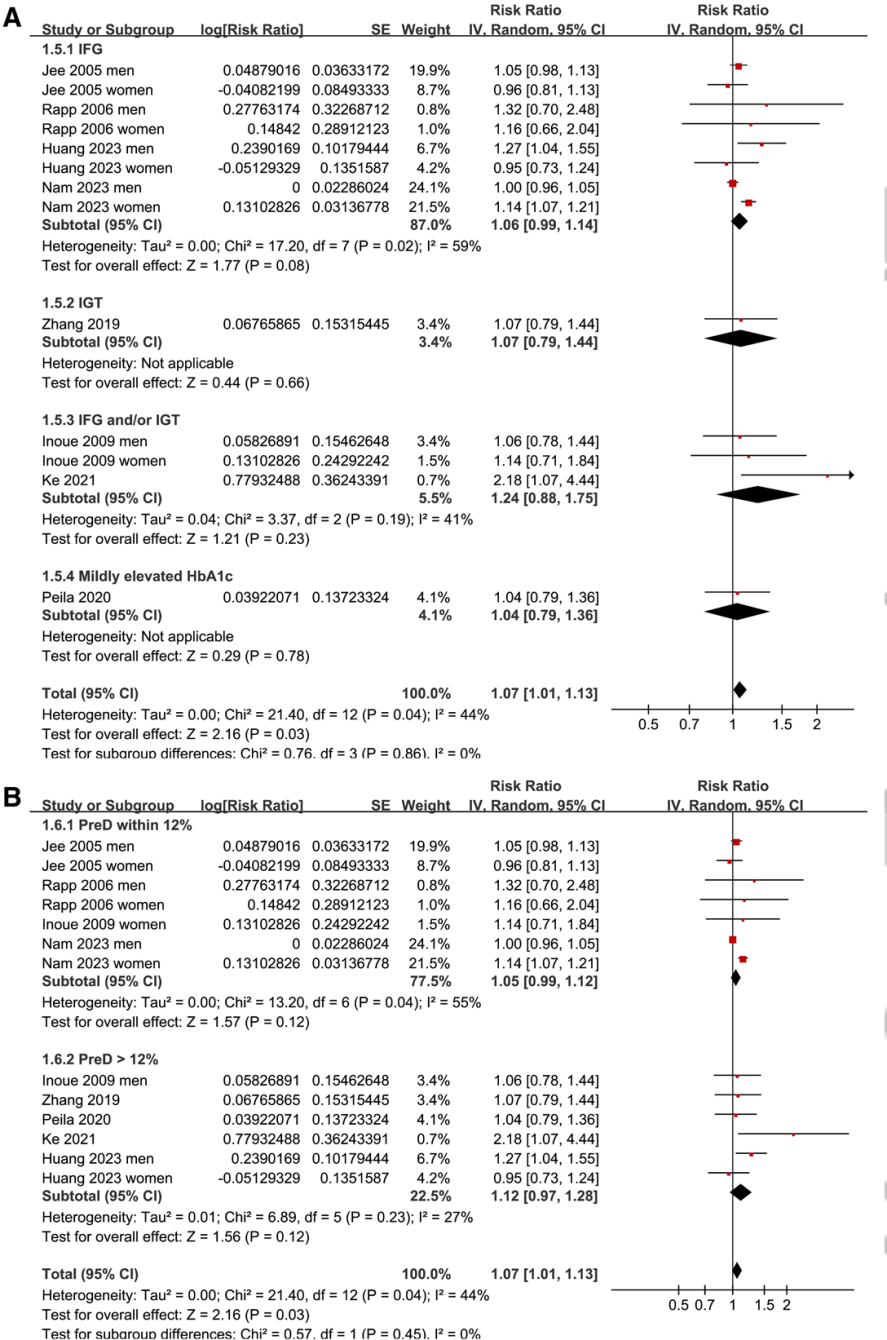
Forest plots for the subgroup analyses of the association between prediabetes and the risk of GC; (A) forest plots for the subgroup analysis according to the definition of prediabetes; and (B) forest plots for the subgroup analysis according to the prevalence of prediabetes in each study. GC = gastric cancer.

**Figure 5.**
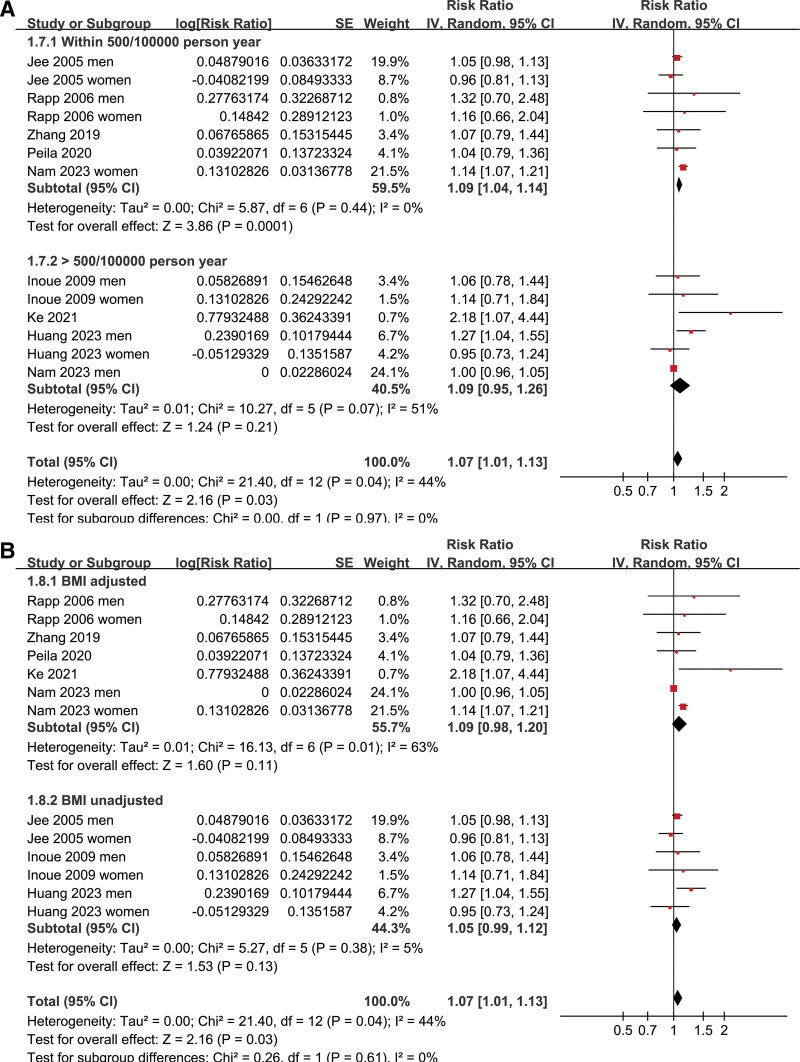
Forest plots for the subgroup analyses of the association between prediabetes and the risk of GC; (A) forest plots for the subgroup analysis according to the incidence of GC in each study; and (B) forest plots for the subgroup analysis according to the adjustment of BMI. GC = gastric cancer.

Pooled results of 13 datasets from the 8 prospective cohort studies^[16–23]^ showed that prediabetes was associated with a mildly increased incidence of GC during follow-up (risk ratio: 1.07, 95% confidence interval: 1.01–1.13, *P* = .03; Fig. 2A) with moderate heterogeneity (I^2^ = 44%). Sensitivity analysis by excluding one dataset at a time showed consistent results (RR: 1.03–1.09, *P* all < .05; Table 3). Results of meta-regression analysis did not suggest that mean age, proportion of men, or follow-up duration did not significantly modify the association between prediabetes and GC (p all > 0.05; Table 4). Subsequent subgroup analyses suggested that the association between prediabetes and the increased risk of GC may not be significantly modified by study country (*P* value of subgroup difference = 0.88; Fig. 2B), mean age of the population (*P* value of subgroup difference = 0.41; Fig. 3A), sex of the participants (*P* value of subgroup difference = 0.65; Fig. 3B), definition of prediabetes (*P* value of subgroup difference = 0.86; Fig. 4A), prevalence of prediabetes of the studied population (*P* value of subgroup difference = 0.45; Fig. 4B), incidence of GC of the population in each study (*P* value of subgroup difference = 0.97; Fig. 5A), or adjustment of BMI in the multivariate analyses (*P* value of subgroup difference = 0.61; Fig. 5B).

**Table 3:** Results of sensitivity analysis.

Dataset excluded	RR (95% CI)	*P* value	*I* ^2^
Jee 2005^[16]^ men	1.07 [1.01, 1.16]	.04	49%
Jee 2005^[16]^ women	1.08 [1.01, 1.15]	0.02	46%
Rapp 2006^[17]^ men	1.07 [1.01, 1.14]	0.04	47%
Rapp 2006^[17]^ women	1.07 [1.01, 1.13]	0.04	48%
Inoue 2009^[18]^ men	1.07 [1.01, 1.14]	0.04	49%
Inoue 2009^[18]^ women	1.07 [1.01, 1.13]	0.04	48%
Zhang 2019^[19]^	1.07 [1.01, 1.14]	0.04	49%
Peila 2020^[20]^	1.07 [1.01, 1.14]	0.04	49%
Ke 2021^[21]^	1.06 [1.01, 1.12]	0.03	37%
Huang 2023^[22]^ men	1.05 [1.01, 1.10]	0.04	38%
Huang 2023^[22]^ women	1.07 [1.01, 1.14]	0.02	47%
Nam 2023^[23]^ men	1.09 [1.03, 1.15]	0.002	14%
Nam 2023^[23]^ women	1.03 [1.01, 1.06]	0.01	11%

**Table 4:** Univariate meta-regression analysis.

Variables	RR for the association between prediabetes and GC		
	Coefficient	95% CI	*P* values
Mean age (years)	0.0024	‐0.0118 to 0.0167	.74
Men (%)	‐0.0002	‐0.0015 to 0.0011	.79
Follow-up duration (years)	‐0.024	‐0.093 to 0.045	.55

CI = confidence interval; GC = gastric cancer; RR = risk ratio.

### 3.4. Publication bias

**Figure 6.**
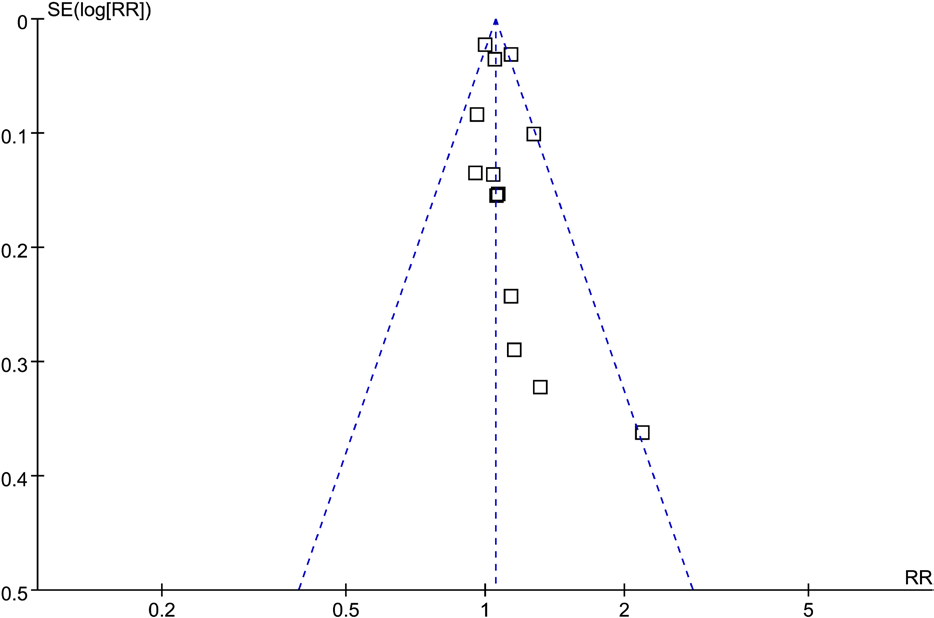
Funnel plots for the publication bias underlying the meta-analysis of the association between prediabetes and the risk of GC. GC = gastric cancer.

Figure 6 illustrates the funnel plots showing the relationship between prediabetes and the risk of developing GC. Upon visual inspection, the plots exhibit symmetry, indicating minimal publication bias. Furthermore, the application of Egger regression tests yielded a low probability of publication bias (*P* = .47).

## 4. Discussion

This meta-analysis integrated data from 13 datasets obtained from 8 prospective cohort studies that were deemed relevant. The findings indicated that prediabetes in the adult population derived from the community may be linked to a slight elevation in the risk of GC. Sensitivity analysis by excluding one dataset at a time showed consistent results. Meta-regression analysis did not suggest that mean age, proportion of men, or follow-up duration did not significantly modify the association between prediabetes and GC. Further analysis of subgroups substantiated the strength of this association, which appeared to be unaffected by various factors, including the country in which the study was conducted, the average age of participants, gender distribution, the definition of prediabetes, the prevalence of prediabetes in the studied population, the incidence of GC in each study, and the adjustment of BMI. Taken together, results of the meta-analysis suggested that compared to people with normoglycemia, a mildly increased risk of GC could be observed in people with prediabetes. These findings further supports that hyperglycemia may be a risk factor of GC, even in a prediabetic state before the diagnosis of diabetes.

Based on our knowledge, there is a scarcity of meta-analyses examining the correlation between prediabetes and the risk of GC. A preliminary meta-analysis encompassing 16 prospective cohort studies indicated that prediabetes is linked to a general heightened risk of cancer.^[15]^ Subsequent subgroup analysis, which incorporated 3 datasets, suggested that prediabetes may also be associated with an increased risk of developing or succumbing to GC/colorectal cancer.^[15]^ However, it was not possible to conduct an analysis solely focused on the incidence of GC due to the limited availability of datasets.^[15]^ In the present meta-analysis, a comprehensive literature search was conducted across 3 widely utilized literature databases, yielding the most recent studies investigating the correlation between prediabetes and the risk of GC. All of the studies included in this analysis were prospective cohort studies, thereby reducing the likelihood of recall and selection biases associated with retrospective designs and enabling the establishment of a longitudinal association between prediabetes and GC. Furthermore, multivariate analysis was employed in all the included studies to examine the correlation between prediabetes and GC, thereby providing support for the possibility that this association is not influenced by potential confounding variables such as age, sex, smoking, and alcohol consumption, among others. Additionally, the results of a series of predefined subgroup analyses indicated that these characteristics did not significantly alter the outcome of the meta-analysis, thus highlighting the strength and reliability of the findings. Specifically, our study reveals that the correlation between prediabetes and the heightened susceptibility to GC is not substantially influenced by the adjustment of BMI. This finding holds significance as recent evidence has established a causal link between obesity and an elevated risk of GC.^[28,29]^ Moreover, prediabetes is closely intertwined with obesity, potentially serving as an underlying mechanism that explains the association between prediabetes and obesity-related malignancies, such as breast cancer.^[30]^ These findings suggest that the association between prediabetes and an increased risk of GC is likely to be independent of obesity.

It remains unclear how prediabetes may contribute to an increased risk of GC. Chronic hyperglycemia has been related to insulin resistance,^[31]^ chronic low-degree systemic inflammation,^[32]^ excessive oxidative stress,^[33]^ and accumulated advanced glycation end-products,^[34]^ which may all participate in the pathogenesis of GC. An early endoscopic study showed that hyperglycemia is associated with gastric dysplasia, supporting an important role of hyperglycemia in the pathogenesis of GC.^[35]^ Moreover, a few preclinical studies have explored the potential molecular mechanisms underlying the role of hyperglycemia in the pathogenesis and progression of GC, such as enhancing the expression of Aquaporin 3,^[36]^ activating enolase 1,^[37]^ upregulating peptidyl–prolyl cis/trans isomerase,^[38]^ and downregulating microRNA-26.^[39]^ The key molecular pathways involved in the role of hyperglycemia in GC development remain to be investigated.

The meta-analysis indicates a higher risk of GC in individuals with prediabetes, leading to important clinical implications for screening and monitoring strategies. This includes the consideration of early screening initiatives, risk stratification based on individual factors, tailored surveillance protocols, integration with glycemic management, patient education and awareness about the increased risk, and the need for further research to better understand the underlying mechanisms linking prediabetes to GC risk. These measures aim to mitigate the burden of GC among prediabetic individuals.

There are also several limitations to the current meta-analysis. First, the number of available datasets included in the meta-analysis is limited, particularly for some subgroup analyses. Accordingly, results of subgroup analyses should be considered as hypothesis generating and interpreted with caution. Second, among all the included studies, GC was confirmed via the data of cancer registry rather than clinical evaluation. Accordingly, we were unable to determine if the association between prediabetes and GC are similar in difference histological type of GC. Studies are warranted to determine if the association between prediabetes and GC varies according to different histological types of the cancer. Moreover, although multivariate analyses were performed in all of the included studies, we were unable to exclude the possibility that the association between prediabetes and GC could be confounded by unadjusted factors, such as dietary and nutritional factors. Furthermore, because the meta-analysis was based on observational studies, the results did not illustrate a causal relationship between prediabetes and increased GC risk. Finally, the studies included primarily focus on community-derived adult populations, which may limit the applicability of the findings to other groups or settings.

In conclusion, the results of the meta-analysis suggest that the presence of prediabetes in community-derived adult populations may be linked to a slightly elevated risk of gastric cancer when compared to individuals with normoglycemia. These findings provide support for the notion that hyperglycemia may serve as a risk factor for gastric cancer, even in the prediabetic stage prior to the diagnosis of diabetes.

## Author contributions

**Conceptualization:** Shenggang Wang, Chong Liu.

**Data curation:** Shenggang Wang, Jiamin Zhao.

**Formal analysis:** Shenggang Wang, Jiamin Zhao, Chong Liu.

**Investigation:** Shenggang Wang, Jiamin Zhao, Chong Liu.

**Methodology:** Shenggang Wang.

**Software:** Shenggang Wang, Chong Liu.

**Supervision:** Chong Liu.

**Validation:** Shenggang Wang, Jiamin Zhao, Chong Liu.

**Visualization:** Shenggang Wang, Jiamin Zhao, Chong Liu.

**Writing – original draft:** Shenggang Wang.

**Writing – review & editing:** Shenggang Wang, Jiamin Zhao, Chong Liu.

## Supplementary Material
