# Supplementary material for: Association between prediabetes and the incidence of gastric cancer: A meta-analysis
Source: Medicine (Baltimore). 2024 Aug 23;103(34):e39411. doi: 10.1097/MD.0000000000039411 (PMC11346863; doi:10.1097/MD.0000000000039411)
Supplement: Supplementary file 1 [file medi-103-e39411-s001.docx]

Search strategy for PubMed, Embase, and Web of Science

("prediabetes" OR "prediabetic" OR "pre-diabetes" OR "pre-diabetic" OR "borderline diabetes" OR "prediabetic state" OR "impaired fasting glucose" OR "IFG" OR "impaired glucose tolerance" OR "IGT" OR "HbA1c"OR "fasting glucose") AND ("gastric" OR "stomach") AND ("neoplasms" OR "cancer" OR "tumor" OR "carcinoma" OR "adenoma" OR "malignancy")
